# Identification and functional analysis of m^6^A in the mammary gland tissues of dairy goats at the early and peak lactation stages

**DOI:** 10.3389/fcell.2022.945202

**Published:** 2022-10-18

**Authors:** Shujun Wang, Lu Zhang, Rong Xuan, Qing Li, Zhibin Ji, Tianle Chao, Jianmin Wang, Chunlan Zhang

**Affiliations:** ^1^ College of Animal Science and Technology, Shandong Agricultural University, Taian, China; ^2^ College of Biological and Agricultural Engineering, Weifang University, Weifang, China

**Keywords:** dairy goats, mammary gland, lactation, MeRIP-seq, m^6^A

## Abstract

N6-methyladenosine (m^6^A) is the most common reversible epigenetic RNA modification in the mRNA of all higher eukaryotic organisms and plays an important role in the regulation of gene expression and cell function. In this study, m^6^A-modified methylated RNA immunoprecipitation sequencing (MeRIP-seq) and transcriptome sequencing (RNA-seq) were used to identify the key genes with m^6^A modification during mammary gland development and lactation in dairy goats. The results showed that m^6^A methylation occurred at 3,927 loci, which were significantly enriched in the 3′ untranslated region (3′UTR) and the termination codon region. In the early stage and peak stage of lactation, m^6^A methylation occurred extensively in mammary tissues, and a total of 725 differentially expressed m^6^A-modified genes were obtained, all negatively correlated with mRNA expression. In addition, Gene Ontology (GO) enrichment and Kyoto Encyclopedia of Genes and Genomes (KEGG) pathway analysis showed that different methylated genes were mainly involved in the growth and apoptosis of mammary epithelial cells through signaling pathways, such as the mitogen-activated protein kinase (MAPK) and phospholipase D pathways, and then affected the development and lactation of mammary gland. All in all, we identified and analyzed the methylation events related to the development and lactation regulation of mammary gland at the early and peak lactation stages, and provided a theoretical basis to reveal the physiological regulatory system of mammary gland development and lactation in dairy goats.

## Introduction

In the 1970s, scientists discovered that m^6^A modification can occur on RNA adenine (A). Subsequent studies showed that m^6^A methylation is not the only modification that exists in the mRNA of prokaryotes, eukaryotes, and viruses; more than 150 posttranscriptional modifications have been revealed in the RNA of all organisms (Dubin and Stollar, 1975; Boccaletto et al., 2018). The molecular functions of m^6^A are diverse but ultimately affect mRNA transcription by regulating splicing, half-life, stability, and translation (Nachtergaele and He, 2018). m^6^A derivatives mediate the posttranscriptional regulation of gene expression to ensure the precise control of multiple biological processes. Currently, studies on m^6^A have been conducted in humans, plants, and yeast (Bodi et al., 2015; Wang M et al., 2020; Hu et al., 2021). In mammals, m^6^A has been investigated in swine, cattle, and cashmere goats (Cao et al., 2020; Wang T et al., 2020; Li et al., 2021). It is mainly involved in the regulation of spermatogenesis, oogenesis, embryonic development, and stem cell pluripotency (Lin et al., 2017; Fan et al., 2019; Ji and Zhang, 2021; Xu et al., 2021).

The mammary gland is one of the unique organs of mammals, which function is to produce and secrete milk to feed offspring (Macias and Hinck, 2012). Its development can be divided into five stages, i.e., embryonic stage, puberty, gestation, lactation, and degeneration, and the developmental process is mainly regulated by hormones, growth factors, and cytokines (Brisken and Ataca, 2015). There are many physiological differences in the mammary gland at different stages of development and lactation. From the early stage to the peak stage of lactation, mammary epithelial cells continue to differentiate, the number of lactating cells increases, lactation activity increases, and the lactation volume gradually increases, reaching a maximum at the peak stage of lactation (Stefanon et al., 2002). Studies on mammary gland development and lactation in dairy goats mostly focus on mRNA (Ji et al., 2019), long noncoding RNAs (lncRNAs) (Ji et al., 2020), and microRNA (Xuan et al., 2020), not on m^6^A. Therefore, in-depth studies of the key genes, signaling pathways, and their regulatory mechanisms in the development of mammary glands in dairy goats are of great value.

The aim of this study was to explore differentially expressed m^6^A-methylated genes in the mammary gland tissues of Laoshan dairy goats during the early and peak lactation stages through methylated RNA immunoprecipitation sequencing (MeRIP-Seq) and to analyze the mechanism of regulation of the development and lactation of mammary gland tissue in the early and peak lactation stages in dairy goats. This study is expected to provide a theoretical basis for the molecular breeding of Laoshan dairy goats.

## Materials and methods

### Animals

The three Laoshan dairy goats used in this study were all from the Qingdao Laoshan dairy goat breeding farm. Mammary gland tissue was collected by surgical procedure after general anesthesia during the early lactation period (postpartum 20 days) and the peak lactation period (postpartum 90 days),respectively. The dairy goats used in the experiment were randomly selected from the group, all healthy, non-inbred individuals, 2 years old, first parity, and similar birth date, weight, and lambing, they were uniformly managed and fed. All experimental animal/procedures were treated/performed in accordance with the guidelines of the Experimental Animal Management Committee of Shandong Agricultural University. Every effort was made to reduce animal suffering during the experiments.

### RNA extraction and quality control

Total RNA was extracted using a Trizol kit (Invitrogen, United States). The integrity of the RNA samples was evaluated using an Agilent 2100 B bioanalyzer (Agilent Technologies, United States). A Nano Photometer spectrophotometer was used to analyze DNA contamination. A Qubit 2.0 fluorometer was used to accurately quantify the RNA concentration used to construct the sequencing library. RNase-free agarose gel electrophoresis was used for visualization.

### Library construction and sequencing

Eukaryotic mRNA from the extracted total RNA was enriched using Oligo (dT) beads, and a Ribo-Zero™ Magnetic Kit (Epicentre, United States) was used to remove rRNA and enrich prokaryotic mRNA. Then, the enriched mRNA fragments were broken into short fragments using fragment buffer, and the RNA was broken into two samples, one of which was used as the input control. The transcriptome sequencing library was constructed to eliminate noise during the capture of methylated fragments. 10 ug total RNA from each sample was enriched respectively with an m^6^A-specific antibody for the library construction; after the m^6^A-modified RNA was captured, the antibody was eluted with magnetic beads to reduce the background noise from nonspecific binding, and the ligation product was subjected to agarose gel electrophoresis, PCR amplification and Illumina Novaseq6000 sequencing. All sequencing work was performed by Gene Denovo Biotechnology Co. Ltd. (Guangzhou, China).

### RNA-seq data analysis

The raw reads obtained from the sequencing included adaptors and low-quality reads. fastp (version 0.18.0) was used to obtain high-quality pure reads (Chen et al., 2018). The specific procedure was as follows: 1) reads containing adaptors were removed; 2) reads containing more than 10% unknown nucleotides (N) were removed; and 3) reads containing more than 50% of low-quality bases (q value ≤20) were removed. HISAT 2.2.4 (Kim et al., 2015) was used to compare the clean data with the reference genome. The matched reads were assembled into transcripts using StringTie v1.3.1 (Pertea et al., 2015; Pertea et al., 2016). For each transcript, RSEM (Li and Dewey, 2011) was used to calculate the FPKM value (fragments per kilobase of transcript per million mapped reads) to quantify expression abundance and change.

### m^6^A-seq data analysis

The raw image data obtained by sequencing were converted into sequence data *via* base calling, which is called raw data and stored in FASTQ file format. To ensure data quality, quality control was performed on the original data to reduce the noise through data filtering and obtain high-quality clean reads for subsequent analysis. HISAT was used to align the clean reads with the reference genome of Capra hircus (version: GCF_001704415.1_ARS1) with default parameters for subsequent analysis. ExomePeak2 (version: 1.0.0) (Meng et al., 2014) was used to perform peak calling in the whole genome, and the threshold was *p* < 0.05. The position information for peaks (RNA regions and sites where m^6^A modification occurs) in the genome, and sequence information for peak regions, were analyzed to screen out peak-related genes. RNA methylation rate = RPM (MeRIP)/RPM (input) was used to calculate the relative methylation rate of each peak, and then exomePeak2 (Meng et al., 2014) was used for differential analysis of the RNA methylation rate for all peaks in the IP group. FDR<0.05 and |log2FC|>1 (Wang Y et al., 2020) were used to screen differential peaks and perform Gene Ontology (GO) enrichment and Kyoto Encyclopedia of Genes and Genomes (KEGG) functional enrichment analysis of differentially expressed peak-related genes.

### Correlation analysis of m^6^A-seq and RNA-seq data

To comprehensively compare the relationship between m^6^A methylation level and gene expression abundance, correlation analysis was performed for m^6^A-seq and RNA-seq data. The peak-related genes were sorted on the basis of their expression levels and divided into 20 equal parts, and the proportion of peak in each part was analyzed. The correlation between expression level and peak enrichment fold change was analyzed using the basic functions of the R package to create a scatter plot of the gene expression-peak enrichment fold change, and the number of genes shared between differentially expressed genes (DEGs) in the transcriptome and differentially methylated genes (DMGs) identified *via* MeRIP-seq were analyzed to find potential inter-omics linked genes. The fold difference was used as the dividing standard to draw a nine-quadrant map to analyze the coregulatory relationship among common DEGs. The default threshold for screening DEGs was |log2FC|>1 (Wang Y et al., 2020). The coregulated genes obtained from the nine-quadrant map were used for subsequent GO and KEGG enrichment analysis to investigate the function of m^6^A-modified mRNA.

### GO and KEGG enrichment analyses

GO (Ashburner et al., 2000) is an internationally standardized gene function classification system that maps DEGs to various terms in the GO database (http://www.geneontology.org/). The number of genes for each term was calculated, and the number of genes with a certain GO function (molecular function, cellular composition, and biological process) were counted. The hypergeometric test was used to find the GO entries that were significantly enriched in the DEGs against the entire reference gene. The p value is calculated using the following formula:

$$P=1-\overset{m-1}{\underset{i=0}{\sum }}\frac{\left(\frac{M}{i}\right)\left(\frac{N-M}{n-i}\right)}{\frac{N}{n}}$$

Where N is the number of genes with a GO annotation; n is the number of DEGs in N; M is the number of genes annotated as a specific GO term; and m is the number of DEGs annotated to a specific GO term. After the calculated p value underwent Bonferroni correction, the corrected-p ≤ 0.05 was used as the threshold to obtain GO terms that were significantly enriched in the DEGs. The main biological functions of DEGs were determined by GO functional significance enrichment analysis.

KEGG (Kanehisa and Goto, 2000) is the main public database for pathways. Pathway significance enrichment analysis was performed using KEGG pathways as the unit, and a hypergeometric test was used to identify pathways that were significantly enriched in DEGs. The calculation formula for the p value is the same as that for the p value of the GO functional significance enrichment analysis, where N is the number of genes with a pathway annotation; n is the number of DEGs in N; M is the number of genes annotated as a specific pathway; and m is the number of DEGs annotated as a specific pathway. Pathways with a Q ≤ 0.05 were defined as pathways that were significantly enriched in differentially expressed proteins.

### Construction of regulatory networks

Genes related to mammary gland development and lactation were selected based on the GO and KEGG annotation results, and gene regulatory networks were constructed using Scytoscape v3.9.1 software (Shannon et al., 2003) and the STRING database (Version 11.5).

## Results

### Comparison of the quality of the sequencing data and the reference genome

In this study, MeRIP-seq was used to identify the m^6^A data (IP) and corresponding mRNA data (input, IN) for m^6^A methylation in dairy goats at the early stage (E-stage, postpartum 20 days) and peak stage (P-stage, postpartum 90 days) of lactation. In the RNA-seq library, 166,972,650 and 160,794,082 raw reads were obtained from the three mammary gland samples in the early and peak stages, of which 165,695,678 and 159,511,700 were clean reads, accounting for 99.24% and 99.2% of the reads, respectively. The Q20% values for the early and peak stages were 97.32% and 97.40% respectively, and the Q30% values were 92.22% and 92.37%, respectively. In the MeRIP-seq library, 15,634,516 and 140,106,744 raw reads were obtained for mammary gland samples from the early and peak stages, of which 152,341,796 and 137,170,868 were clean reads, accounting for 97.44% and 97.9% of the reads, respectively. The Q20% values for the early and peak stages were 92.62% and 93.35%, respectively, and the Q30% values were 86.05% and 87.09%, respectively (Table 1).

**TABLE 1:** Comparison of the quality of sequencing data and the reference genome between the two libraries.

Sample	Raw data	Clean reads	Q20%	Q30%	GC%	Unique mapped reads	Multiple mapped reads	Total mapped
E1-IN	49336232	48723832 (99.01%)	6989937247 (97.26%)	6613256954 (92.02%)	3645518978 (50.72%)	41781542 (85.75%)	3549962 (7.29%)	45331504 (93.04%)
E1-IP	47274568	45934328 (97.25%)	2021227037 (92.21%)	1871690851 (85.39%)	1137970055 (51.92%)	25455216 (55.42%)	9434574 (20.54%)	34889790 (75.96%)
E2-IN	52823280	52215686 (99.02%)	7497513916 (97.32%)	7095665407 (92.10%)	3798320528 (49.30%)	45085065 (86.34%)	4680842 (8.96%)	49765907 (95.31%)
E2-IP	50759632	49339766 (97.25%)	2216090699 (92.50%)	2054835901 (85.77%)	1241473438 (51.82%)	30472303 (61.76%)	8603775 (17.44%)	39076078 (79.20%)
E3-IN	64813138	64356886 (99.58%)	9254754450 (97.39%)	8792123099 (92.53%)	4696932492 (49.43%)	55296223 (85.92%)	6533475 (10.15%)	61829698 (96.07%)
E3-IP	58311116	56966536 (97.76%)	2373500143 (93.15%)	2216974301 (87.00%)	1291671026 (50.69%)	36019943 (63.23%)	10062207 (17.66%)	46082150 (80.89%)
P1-IN	50993434	50524106 (99.25%)	7295972140 (97.32%)	6901424197 (92.06%)	3711262517 (49.50%)	43640296 (86.38%)	4544886 (9.00%)	48185182 (95.37%)
P1-IP	44199300	43141986 (97.65%)	1688812739 (93.05%)	1577322072 (86.91%)	935110181 (51.52%)	27756879 (64.34%)	6412478 (14.86%)	34169357 (79.20%)
P2-IN	49823380	49215396 (99.11%)	7017856509 (97.28%)	6651148714 (92.20%)	3724866910 (51.63%)	43060635 (87.49%)	3391325 (6.89%)	46451960 (94.39%)
P2-IP	49680806	48636994 (97.97%)	2206924336 (93.49%)	2056514036 (87.12%)	1238363897 (52.46%)	29752521 (61.17%)	9099850 (18.71%)	38852371 (79.88%)
P3-IN	59977268	59418754 (99.24%)	8522800666 (97.60%)	8108542674 (92.85%)	4263050443 (48.82%)	50915336 (85.69%)	6397019 (10.77%)	57312355 (96.45%)
P3-IP	46226638	45314946 (98.07%)	2087434412 (93.51%)	1947410259 (87.24%)	1164951525 (52.19%)	31992607 (70.60%)	5985301 (13.21%)	37977908 (83.81%)

IN, input; IP, m^6^A; E repsents the early stage, E1, E2 and E3 repsents the different libraries.

The P repsents the peak stage, P1, P2 and P3 repsents the different libraries.

**FIGURE 1:**
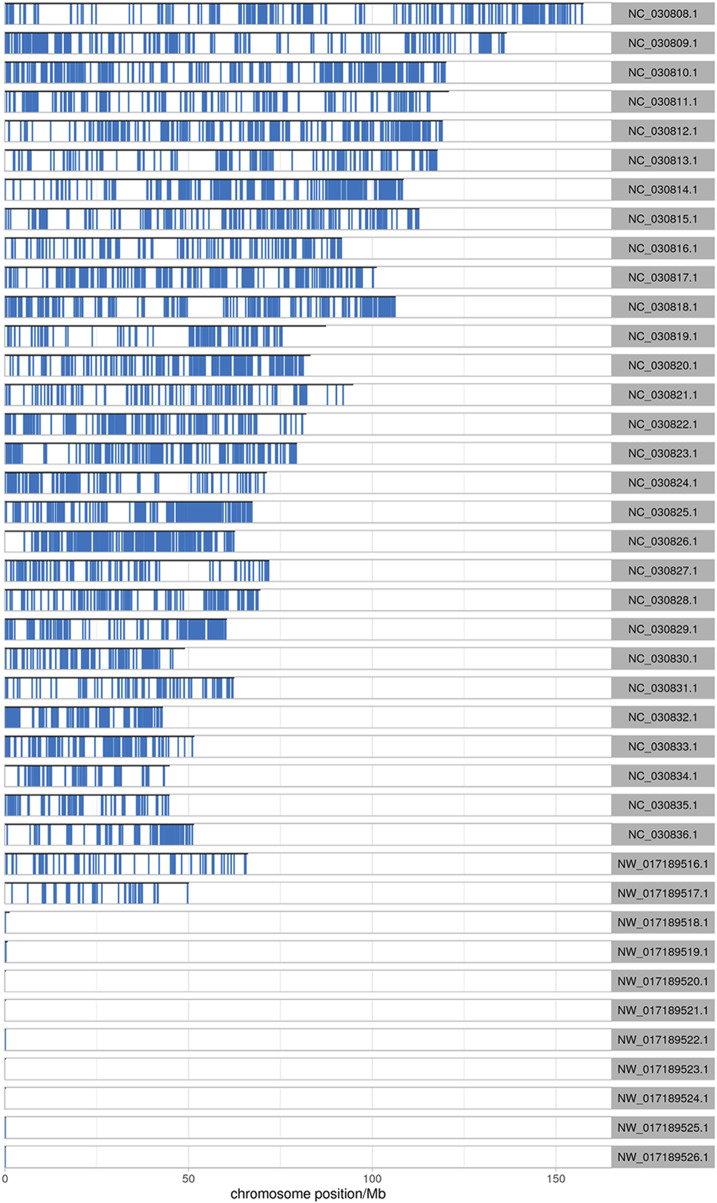
Distribution of reads on chromosomes. The abscissa is the chromosome locus (Mb), and the ordinate is the chromosome ID.

After comparing the reads with the reference sequences, the alignment rate of valid reads for replicated samples of dairy goat mammary gland tissue in the early stage in the RNA-seq library was 93.04%–96.08%, of which the single alignment rate was 85.75%–86.34% and the multiple alignment rate was 7.29%–10.15%. The alignment rate of valid reads for the replicated samples of dairy goat mammary gland tissue in the peak stage was 94.39%–96.46%, of which the single alignment rate was 85.69%–87.49% and the multiple alignment rate was 6.89%–10.77%. In the MeRIP-seq library, the alignment rate of the valid reads for the replicated samples of dairy goat mammary gland tissue in the early stage was 75.96%–80.89%, of which the single alignment rate was 55.42%–63.23% and the multiple alignment rate was 17.44%–20.54%. The alignment rate of valid reads in the replicated samples of dairy goat mammary gland tissue in the peak stage was 79.2%–83.81%, of which the single alignment rate was 61.17%–70.60% and the multiple alignment rate was 13.21%–18.71% (Table 1). The most reads for different samples were distributed on the NC _030808.1 chromosome (Figure 1).

### Identification of m^6^A modification sites and motif analysis

**FIGURE 2:**
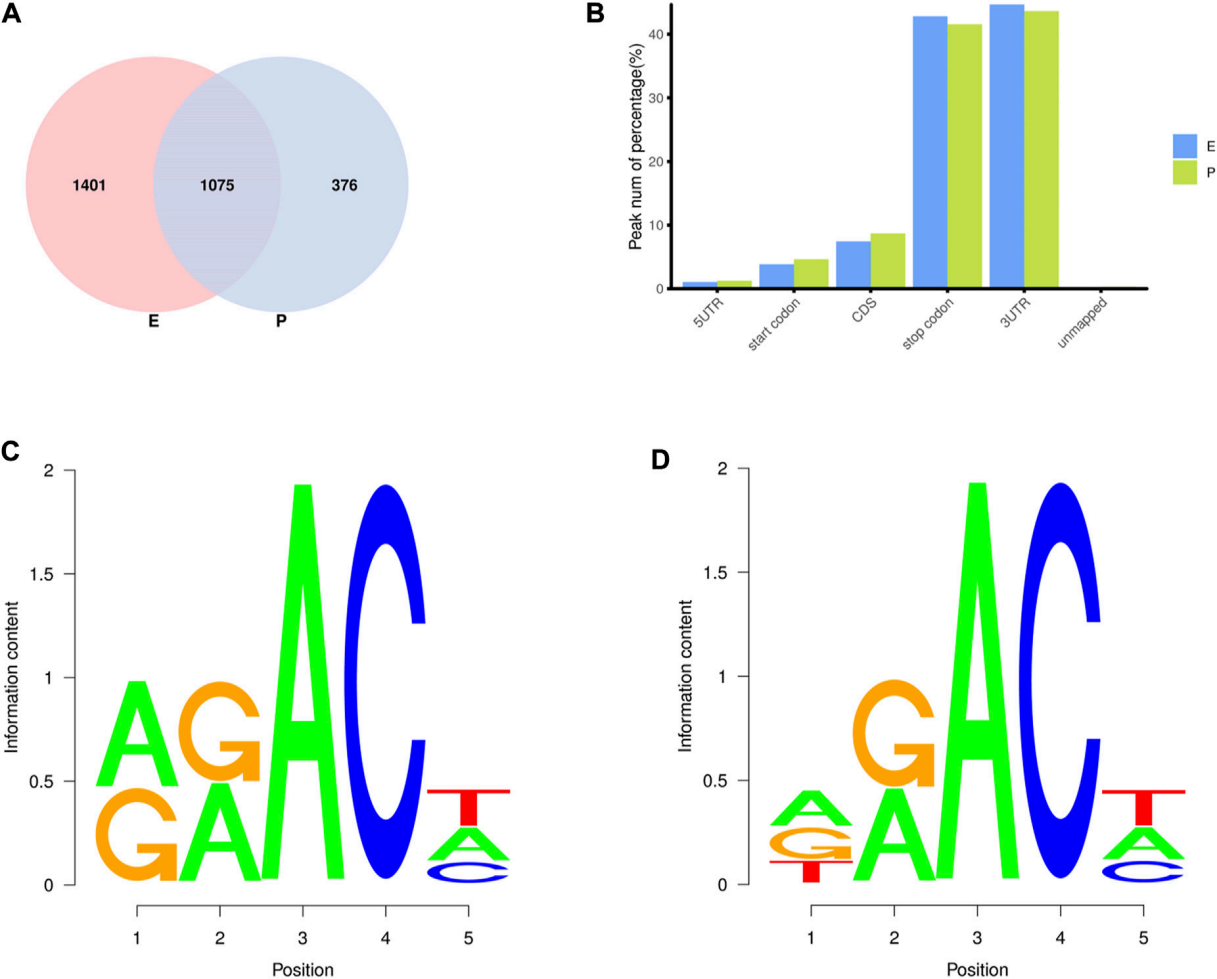
Regional distribution and motif sequence of m^6^A modifications on transcripts. **(A)** Peak distribution of m^6^A modifications in early and peak lactation. **(B)** The distribution of m^6^A in transcripts **(C)** and **(D)** Two common motifs with the highest m^6^A abundance. E represents the early lactation, P represents the peak lactation, the following are the same.

In the two lactation periods, 2,476 peaks were identified during the early stage of lactation, and 1,451 peaks were identified at the peak stage (Figure 2A). To understand the degree of m^6^A modification in genes and to compare the changes in m^6^A gene modification in the two periods, the priority regions of peak gene distribution were analyzed.

The results showed that peaks were significantly enriched in the 3′ untranslated region (3′UTR, 44.67%) and the termination codon region (42.81%), followed by the coding DNA sequence (CDS, 7.43%) and initiation codon region (3.84%) (Figure 2B), these findings are consistent with the results of previous studies on m^6^A modification such as pigs and goose (Cao et al., 2020; Xu et al., 2021). These results indicate that m^6^A modification presents different distribution patterns on different gene functional elements, which indicates that m^6^A is involved in the regulation of gene function, which may have unique functions related to mammary gland development and lactation. In previous studies, researchers found that the m^6^A modification site was often accompanied by motif sequences, e.g., 5′-DRACH-3′ and 5′-RRACH-3' (D = G/A/U, R = G/A, H = A/U/C) (Dominissini et al., 2012; Meyer et al., 2012). This study found that 96.36% sequences contained target motifs (Table 2). The motif sequences with the highest frequency were GGACT (10.55%) (Figure 2C) and AAACA (10.13%) (Figure 2D).

**TABLE 2:** The motif sequences of m^6^A peaks and their proportions in the two lactation stages.

	Motif	*p*-value	% Of target	% Of background
E-IP vs. E-input	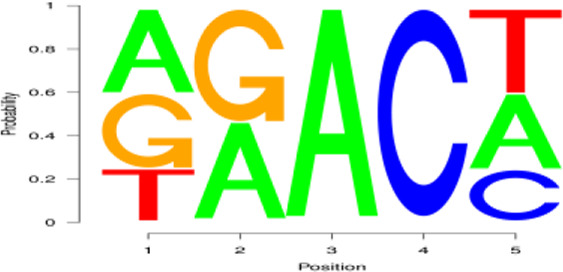	1e-7	97.94	95.76%
	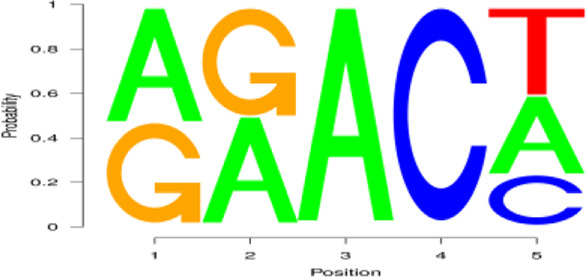	1e-10	95.56	91.77%
P-IP vs. P-input	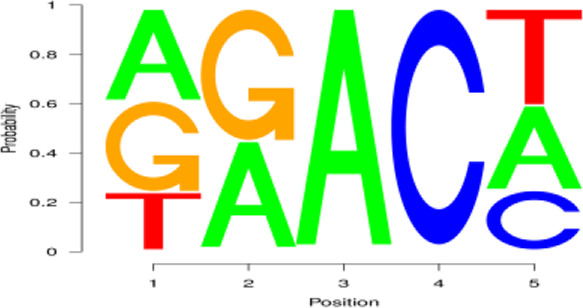	1e-4	97.11	94.70%
	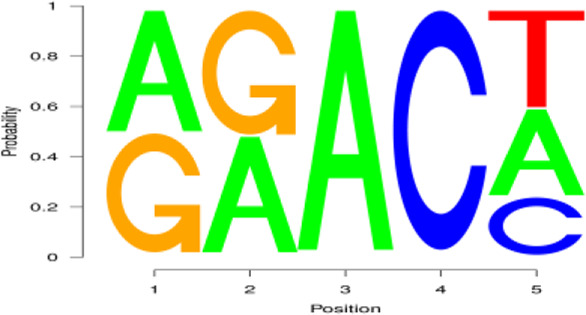	1e-9	94.83	89.83%

Note: Count the frequency (RRACH, DRACH) distribution of specific motifs in all peaks in two periods, and use homer to construct an averaged base frequency matrix for each motif for enrichment analysis.

### RNA-seq gene identification and functional analysis

**FIGURE 3:**
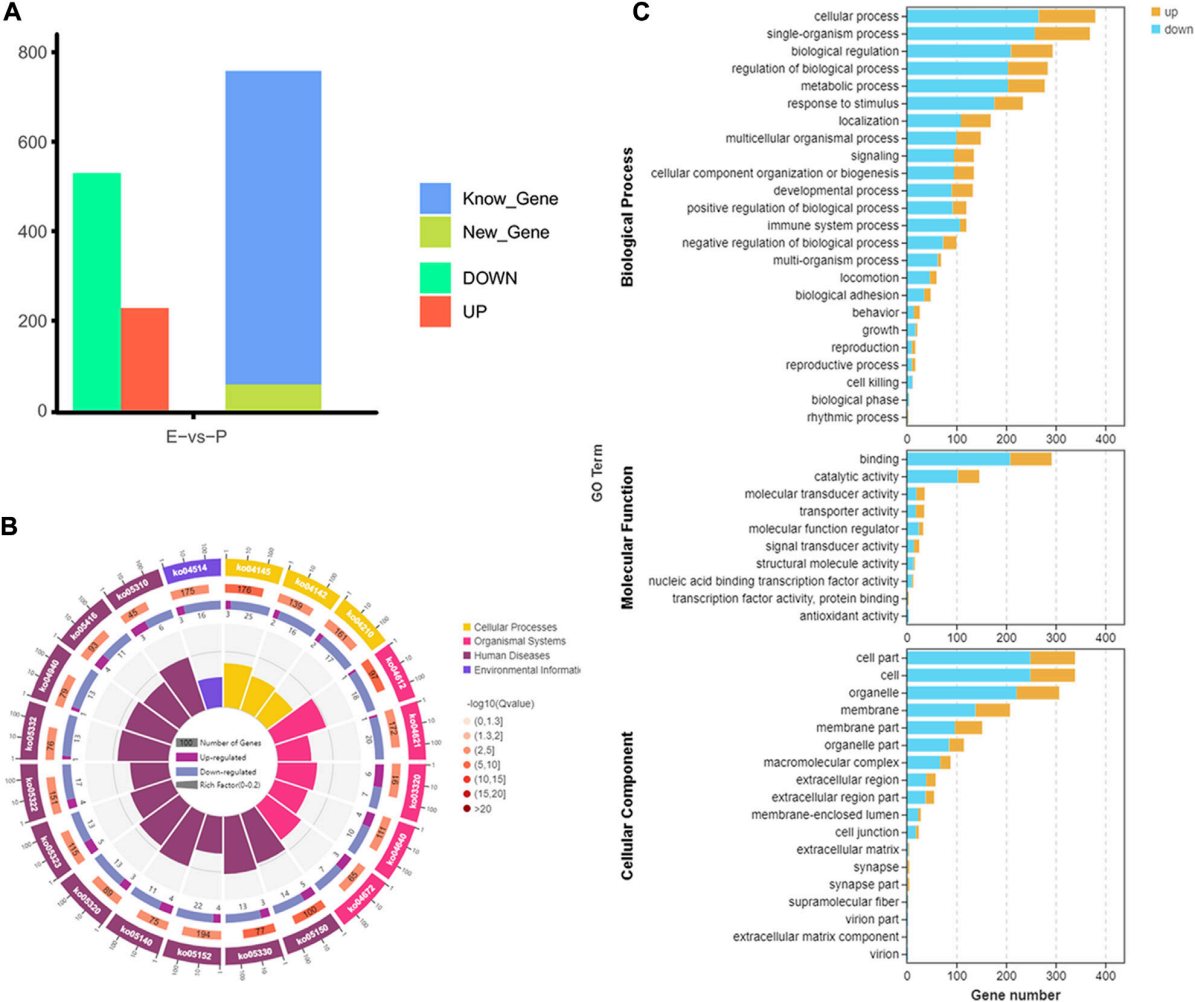
Analysis of gene expression and function in dairy goats during early and peak lactation. **(A)** Analysis results of gene identification at different lactation stages. **(B)** KEGG pathway analysis of DEGs. From inside to outside: the first circle—the top 20 KEGG terms enriched, the coordinates of gene number in the outside circle, and different colors represent different A class; the second circle—the number of genes, different colors represent different Q value, the smaller Q value, the more red color; the third circle—bar graph of gene numbers, dark purple represents the number of upregulated genes, and light purple represents thenumber of downregulated genes; the fourth circle—the rich factor value of each pathway. **(C)** GO annotation analysis results of DEGs.

From the early to peak stages of lactation, a total of 21,518 genes were identified, including 20,606 known genes and 912 new genes. Among the 758 DEGs screened using FDR<0.05 and |log2FC|>1, 228 genes were upregulated, and 530 genes were downregulated during the peak stage of lactation (Figure 3A).

GO enrichment analysis indicated that 553 DEGs were annotated into 54 GO terms, including 150 upregulated DEGs and 394 downregulated DEGs. Among them, 444 DEGs were annotated to 17 cell components, which were mainly distributed in cells, cell parts, organs, and organelles. 471 DEGs were annotated to 23 biological processes, mainly involved in biological regulation, cellular processes, metabolic processes, and single organs. A total of 385 DEGs were annotated to 10 molecular functions, mainly related to binding, catalytic activity, and transport activity (Figure 3C). In the KEGG enrichment analysis, 758 DEGs were involved in four major KEGG pathways, which mainly involved cellular processes (162 genes), environmental information processes (202 genes), genetic information processes (44 genes), and metabolism (180 genes), and were involved in 40 secondary KEGG pathways, including cell growth and apoptosis, cell viability, signal transduction, transport, and catalysis (Figure 3B).

### Identification and functional analysis of the MeRIP-seq peaks

**FIGURE 4:**
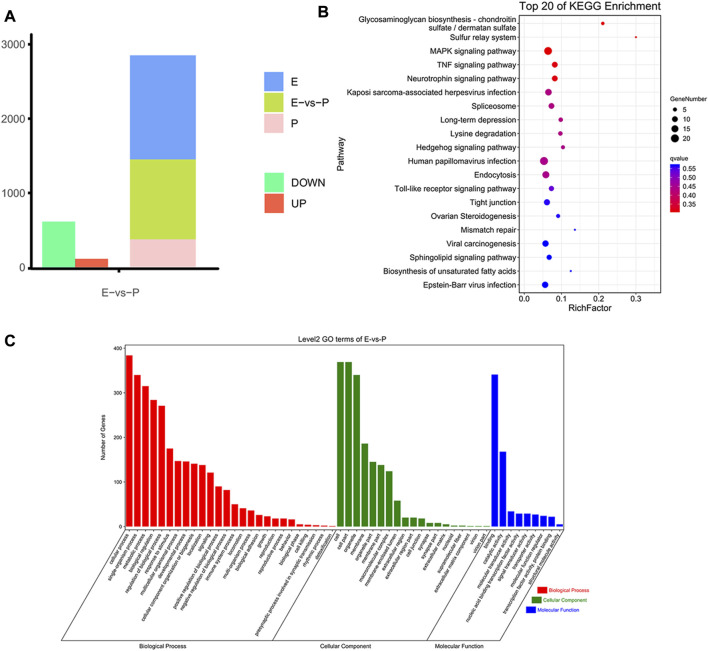
Identification and functional analysis of m^6^A peaks. **(A)** Peak identification during different lactation stages. **(B)** Enrichment results for the top 20 DMGs pathways. **(C)** GO annotation enrichment results for DMGs.

To analyze m^6^A modification in different stages of lactation, MeRIP-seq was used to identify the m^6^A peaks during the early and peak stages of lactation. In the early stage, there were 1,401 unique peaks, and in the peak stage, there were 376 unique peaks; the common peaks in the two stages were 1,075 (Figure 2A). After screening, 725 differential peaks were obtained, of which 112 were upregulation events and 613 were downregulation events during the peak stage of lactation (Figure 4A), which distributed in 720 DMGs (Supplementary Table S1).

GO enrichment analysis of the DMGs indicated that in the three libraries, 553 DMGs were annotated into 54 GO terms: 455 DMGs were annotated to 19 cell components, distributed in cells, cell membranes, cell parts, organs, and organelles; 460 DMGs were annotated to 26 biological processes, involving biological regulation, cellular processes, single biological processes, multicellular biological processes, and reproductive processes; and 429 DMGs were annotated to nine molecular functions, involving binding, catalytic activity, transport activity, molecular function regulation, molecular structure activity, and molecular sensor activity (Figure 4C, Supplementary Table S2.

The functional classification of DMGs was obtained by KEGG pathway analysis. Among the DMGs, 349 were involved in six major KEGG pathways, involving cellular processes (94 genes), environmental information processes (96 genes), genetic information processes (73 genes), human diseases (121 genes), organic systems (105 genes), and metabolism (82 genes). Thirty-nine secondary KEGG pathways were involved, including cell growth and apoptosis, cell viability, membrane transport, signal transduction, signal molecule interaction, transport, and decomposition. Among the 284 pathways analyzed, 24 significantly enriched pathways were identified, including the MAPK signaling pathway, spliceosome signaling pathway, Hedgehog signaling pathway, tight junction signaling pathway, and NF-kappa B signaling pathway et al. The pathways were mainly involved in biological processes such as mammary epithelial cell proliferation and apoptosis (Figure 4B, Supplementary Table S3).

### Correlation analysis of MeRIP-seq and RNA-seq data

**FIGURE 5:**
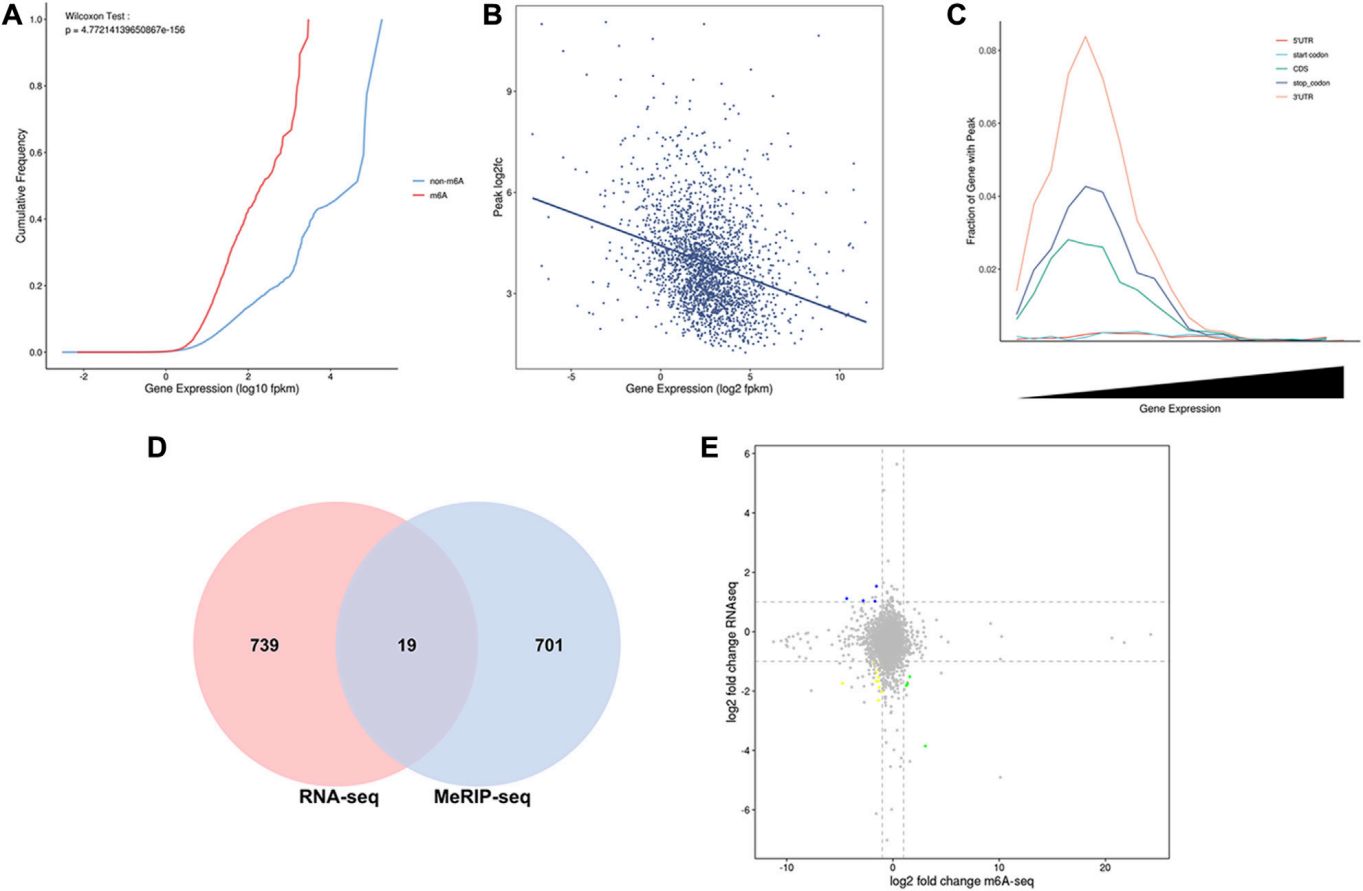
Combined MeRIP-seq and RNA-seq analysis. **(A)** Cumulative curve for gene expression with/without m^6^A modification. The red line represents the gene set with m^6^A peak signal, and the blue line represents the gene set without m^6^A peak signal. **(B)** Relation of gene expression and peak enrichment fold change **(C)** Peak distribution in different gene elements and expression abundance. **(D)** Venn diagram of differential gene distribution in methylomics and transcriptomics **(E)** Nine-quadrant plot of DEGs with differential peaks. The horizontal axis is the fold difference (log_2_) in m^6^A peak abundance, and the vertical axis is the fold difference (log_2_) of gene expression abundance in the transcriptome. Blue dots represent upregulated genes and downregulated m^6^A genes, yellow dots represent downregulated genes and downregulated m^6^A genes, and green dots represent downregulated genes and upregulated m^6^A genes.

In the intragroup association analysis, 2,240 genes were modified by m^6^A methylation during the early stage, and 1,343 genes were modified by m^6^A methylation in the peak stage. According to the cumulative curve, the expression level of genes modified *via* methylation was low under the same cumulative frequency of m^6^A methylation (Figure 5A). Based on the scatter plot of gene expression-peak enrichment fold change, the m^6^A methylation level was negatively correlated with gene expression abundance, i.e., the peak enrichment of relatively highly expressed genes was relatively low (Figure 5B). Through the analysis of the proportion of peaks in different gene elements, it was found that each element exhibited a nonmonotonic functional relationship pattern. When gene expression abundance reached a certain level, the proportion of peaks showed a downward trend as gene expression continued to increase (Figure 5C). In the combined analysis of DEGs and DMGs, 720 DMGs were identified, of which 19 genes were present in the transcriptome (Figure 5D, Supplementary Table S4).

To visually represent the coexpression of genes and m^6^A, we analyzed the nine-quadrant plots and found that 79% of the genes (15 of 19) were downregulated in the differentially expressed m^6^A-modifying genes (Figure 5E). Among them, seven genes are related to mammary gland development and lactation, including three hypomethylated and upregulated genes (*COLGALT2*, *IL20RA*, *PRKG1*), two hypermethylated and downregulated genes (*LOC102185917*, *GPR132*), two hypomethylated and down regulated genes (*GADD45G*, *RGS10*).

### Functional analysis of differential genes enriched peak in two lactation stages

**FIGURE 6:**
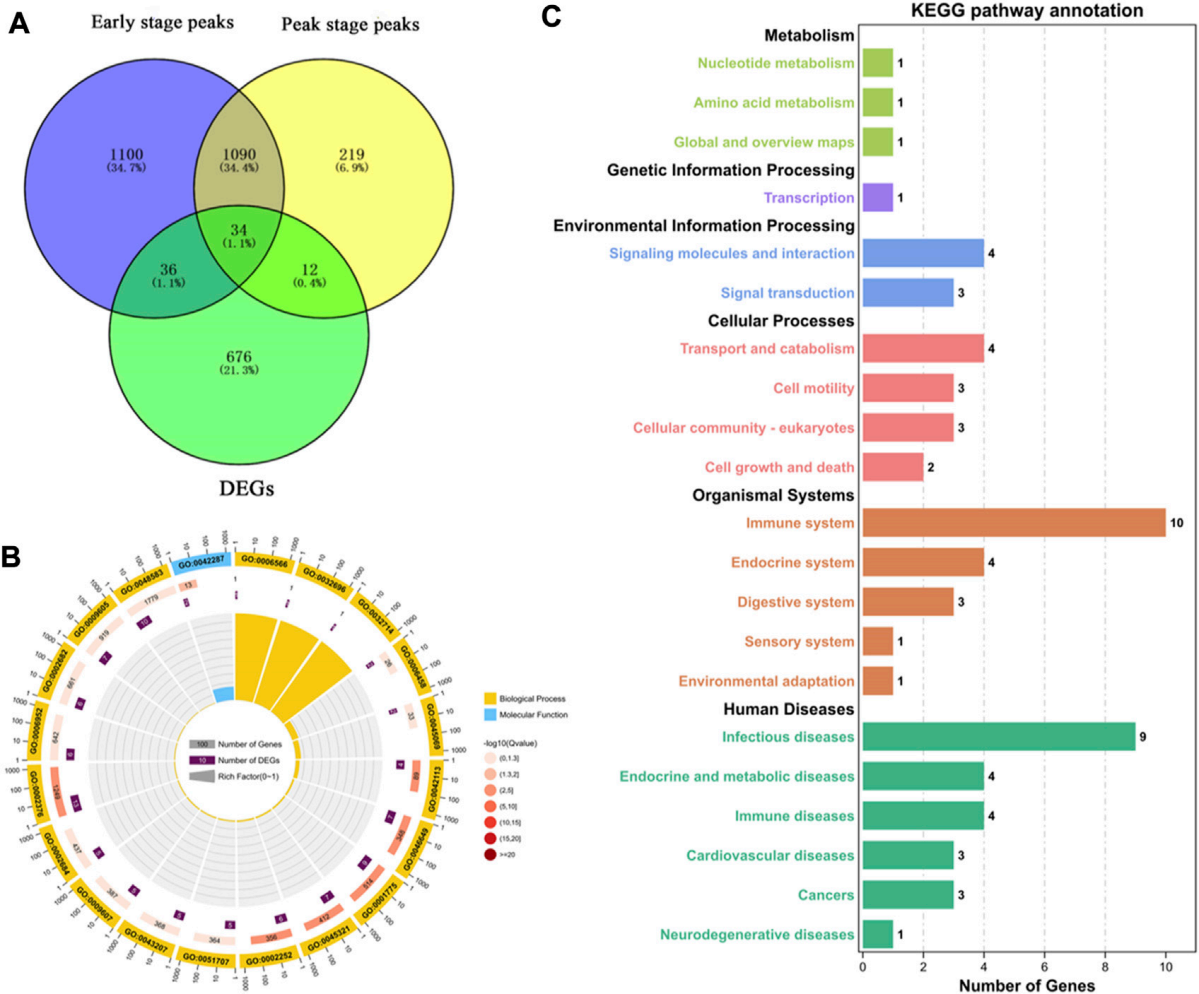
Functional analysis of peak enriched DEGs during early and peak lactation stages. **(A)** Venn diagram of peak distribution in differential expression genes between early and peak lactation stages. **(B)** GO enrichment analysis of the 34 common peak DEGs. **(C)** KEGG enrichment analysis of the 34 common peak DEGs.

To more accurately analyze the relationship between the transcriptome and m^6^A methylation, this study combined analysis of DEMs enriched peaks and the DEGs in the early and peak stages (Figure 6A), found that the peaks in early stage was distributed among 70 DEGs, and in the peak stage the peaks was distributed in 46 DEGs, 36 DEGs were existed uniquely in the early stage, and 12 DEGs were for peak stage uniquely.

In addition, through analysis, it was found that there were 34 genes in common between the differential peak-related genes and the differential transcriptome genes in the two periods, and GO and KEGG enrichment analyses were performed (Supplementary Table S5). The top 20 GO terms were enriched in biological processes and molecular functions, which were mainly concentrated in the regulation of biological processes, cell apoptosis, cell growth processes, cellular components or biogenesis, signal transduction, etc., involving 22 genes (*ISG15*, *ISG20*, *TBX21*, *MALT1*, *SLC27A1*, *ACTB*, *TNFRSF21*, TNFAIP8L2, *VEGFC*, *PLTP*, *ANP32A*, *SLA2*, *TBC1D10C*, *CD8B*, *ITM2C*, *FGD3*, *TMSB4X*, *TUBB*, *RGS1*, *LOC102174841*, *PTMA*, and *PFDN4*) (Figure 6B). In the KEGG enrichment analysis, the relevant pathways were mainly enriched in cellular processes, environmental information processes, gene information processes, and organ systems, including cell transport and catabolism, cell viability, and interactions of signaling molecules, involving phagosomes (*TUBB*, *ACTB*, *LOC102180664*, and *LOC102185917*), cell adhesion molecules (*LOC102180664*, *CD8B*, *LOC102185917*), the PPAR signaling pathway (*SLC27A1* and *PLTP*), glycine, serine, and threonine metabolism (*LOC102174841*), insulin resistance (*SLC27A1*), apoptosis (*ACTB* and *LOC102185917*), the MAPK signaling pathway (*VEGFC*), and the PI3K-Akt signaling pathway (*VEGFC*) (Figure 6C).

### Mammary gland development and lactation regulatory network

**FIGURE 7:**
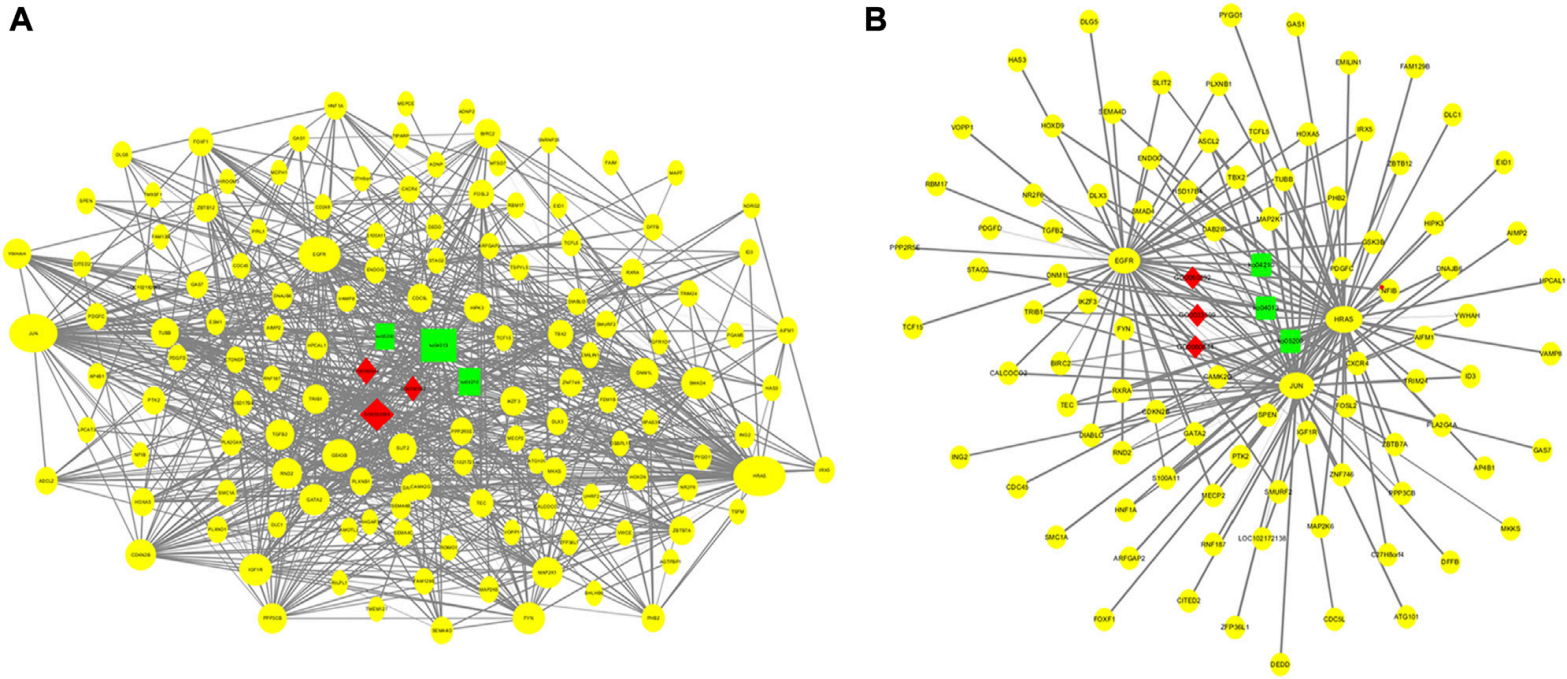
Regulation networks related to mammary gland development and lactation of dairy goats. **(A)** Interaction networks of 150 coexpressed genes. **(B)** Regulation networks of core gene module.

Using the GO and KEGG annotation results, 150 genes that directly annotated mammary gland development and lactation were selected from the 2,468 common genes obtained from the two groups (Figure 7A). These genes are mainly involved in mammary gland formation (GO: 0060592), mammary epithelial cell proliferation (GO: 0033599), mammary gland epithelial cell differentiation (GO: 0060644), and biological processes involved in mammary gland development (GO: 0003006). They are involved in KEGG pathways, such as cancer (ko05200), the MAPK signaling pathway (ko04013), cell apoptosis (ko04210), and the PI3K-Akt signaling pathway (ko04151). Based on the interaction analysis of coexpressed genes in STRING database, consisting of 89 nodes and 378 edges, the core genes that showed the most interactions were *HRAS*, *JUN*, and *EGFR* (Figure 7B).

## Discussion

Methylation modification is an important means of regulating gene expression in epigenetics and also the earliest epigenetic modification discovered. m^6^A methylation is the most conserved and extensive RNA modification in living organisms (Rengaraj et al., 2021). Studies on m^6^A have been conducted in humans, viruses, fruit flies, plants, and yeast. (Bodi et al., 2015; Wang M et al., 2020; Hu et al., 2021). In mammals, only swine, cattle, and cashmere goats have been studied. (Cao et al., 2020; Wang T et al., 2020; Li et al., 2021). However, there have no studies on m^6^A methylation in dairy goats to date, therefore, m^6^A methylation and its mechanism during mammary gland development and lactation in dairy goats are still unknown. At present, numerous studies have shown that m^6^A widely involved in spermatogenesis, oogenesis, skin hair follicle morphogenesis, embryonic development, stem cell pluripotency, and myoepithelial cell differentiation, etc. (Luo et al., 2014; Dai et al., 2018; Hui et al., 2022). m^6^A may also play a crucial role in mammary gland development and lactation of dairy goats.

In this study, coimmunoprecipitation sequencing and general transcription sequencing data were combined to analyze the correlation between m^6^A modification and the expression of mammary gland development and lactation-related genes based on mRNA in the mammary gland tissue of dairy goats. Previous studies have found that m^6^A modification characteristics and patterns are highly consistent in the same species but different in various species (Dominissini et al., 2012; Meyer et al., 2012; Wang A et al., 2021). Based on this technology, we investigated the characteristics and patterns of m^6^A modification, including the degree of m^6^A modification, the distribution position of m^6^A in the transcript, and the m^6^A methylation sequence motif, in the mRNA transcriptome of dairy goats. During mammary gland development and lactation, there were a large number of m^6^A methylation modifications in mammary gland tissue, including 2,476 peaks identified during the early lactation stage and 1,451 peaks identified during the peak lactation stage. In addition, the abundance of m^6^A in the 3′UTR was higher, a finding that is consistent with the abundance pattern of m^6^A in the skin tissue of Liaoning cashmere goats (Wang Y et al., 2021). It was reported that m^6^A peaks are significantly enriched in the CDS and initiation codons (Xu et al., 2021); however, the distribution pattern of m^6^A in the goat methylation group was different from that in goose (Xu et al., 2021), *Bombyx mori* (Li et al., 2019), *mice* (Meyer et al., 2012), and *Arabidopsis* (Luo et al., 2014; Duan et al., 2017), indicating that the distribution pattern of m^6^A is species specific.

Based on the combined analysis of DEGs in transcriptomes and differential peaks, 24 DEGs with m^6^A methylation modifications were identified in this study, all of which were associated with mammary gland development and lactation in goats. These data indicate that there are dynamic changes in the regulation of important processes by m^6^A during mammary gland development and lactation. Similarly, dynamic changes in the m^6^A modification in the follicular selection process of chickens (Fan et al., 2019), different skin tissues of Liaoning cashmere goats (Wang T et al., 2020), and different stages of porcine follicular development in swine (Cao et al., 2020) have also been observed. Among the hypermethylated and downregulated genes in the differentially coexpressed DEGs and DMGs in the combined analysis, the proton-sensing G protein-coupled receptor *GPR132* activate signals and transduce signals into cells by lowering pH (Weiß et al., 2017), and its homolog, *GPR68*, promotes apoptosis and inhibits the proliferation of goat mammary epithelial cells (Zhu et al., 2021). In addition, *PRKG1*, the hypomethylated and upregulated protein kinase, was negatively correlated with the expression of placental-associated miR-517a-3p before and after delivery (Kambe et al., 2014), indirectly regulating mammary gland development and lactation.

m^6^A is a chemical marker associated with transcript degradation (He et al., 2017). High levels of m^6^A modification may endow transcripts with higher stability at lower transcription levels or provide stronger signals for reader proteins, thereby more effectively exerting biological functions (Niu et al., 2013; Wang et al., 2014). In this study, approximately 15% of m^6^A-modified genes had 2 m^6^A modification sites, and approximately 3% of m^6^A-modified genes had 3 m^6^A modification sites, which may also increase RNA stability or the probability of being recognized by reader proteins. These results all indicate that m^6^A modification plays a posttranscriptional regulatory role in the mammary gland transcriptome of dairy goats. To elucidate the possible mechanisms underlying the involvement of differentially coexpressed genes in mammary gland development and lactation regulation, GO and KEGG enrichment analyses were performed. Cells, organelles, and cellular parts were annotated as cellular components; cellular processes, signal transduction, metabolic processes, and biological regulation were annotated as molecular functions; and binding and catalytic activation were annotated as biological processes. For the KEGG pathway analysis, the cancer pathway, the PI3K-Akt signaling pathway, and the MAPK signaling pathway were the main enriched metabolic pathways.

Based on the GO and KEGG pathway analysis results, 150 genes related to mammary gland development and lactation were subjected to an interaction analysis of coexpressed genes. The core genes that showed the most interactions in the network were *HRAS*, *JUN*, and *EGFR*. The p21 protein encoded by the *HRAS* proto-oncogene induces the invasive phenotype of human mammary epithelial cells and plays an important role in the development of breast cancer (Moon et al., 2000). Curcumin can inhibit the signal transduction of HRAS-transformed mammary epithelial cells (HRAS MCF10A) to reduce the incidence of breast cancer (Hahn et al., 2018), thereby promoting mammary gland development and lactation. *JUN* (AP-1 transcription factor subunit) proto-oncogenes include *c-Jun*, *JunB*, and *JunD*. AP-1 is involved in the proliferation and differentiation of lymphocytes, osteoblasts, and keratinocytes (Elkeles et al., 1999; Hess et al., 2004). *JunB* inhibits cell proliferation by activating the expression of p16 (*INK4a*). Furthermore, *JunB* is a negative regulator of cell proliferation (Passegue and Wagner, 2000). Therefore, the *JUN* gene may regulate mammary gland cell apoptosis. Studies have found that c-Jun N-terminal kinase (*JNK*) can regulate the proliferation of mammary gland cells and lactoprotein synthesis in dairy cows by activating Tudor-SN (Ao et al., 2021). *EGFR* is a member of the epidermal growth factor receptor (*HER*) family. Studies have found that *EGFR* promotes adhesion between mammary gland cells and regulates the growth and differentiation of human mammary epithelial cells (Mukhopadhyay et al., 2013). *EGFR*, at concentrations ranging from 12.5 to 50 ng/ml, facilitates the proliferation of mammary epithelial cells in dairy goats, and activation of the EGFR-mediated signaling pathway promotes the survival of mammary epithelial cells in dairy goats (Huang et al., 2020). Therefore, the data obtained in this study provide a basis for future studies on the role of m^6^A methylation in the development of mammary glands in dairy goats.

## Conclusion

In summary, this study revealed the differences in the transcription and methylation levels of genes in mammary gland tissue between the early and peak stage of lactation and explored their regulation in mammary gland development and lactation function. The proportion, distribution and motif of m^6^A gene modification in the mRNA transcriptome of mammary gland tissue from dairy goats were consistent with the pattern of m^6^A modification in the same species; the level of m^6^A modification in mammary gland tissue was highly negatively correlated with the abundance of modified transcripts. The genes that were modified by m^6^A at both stages were mainly involved in the regulation of the proliferation and differentiation of mammary gland epithelial cells and the development of mammary gland tissue. Among 150 genes closely related to mammary gland development and lactation, *HRAS*, *JUN*, and *EGFR* were most likely to play a key role in regulating mammary gland development and lactation. This study can provide a theoretical basis for the molecular mechanism of mammary gland development and lactation regulation in dairy goats.

## Data Availability

The datasets presented in this study can be found in online repositories. The names of the repository/repositories and accession number(s) can be found below: GEO database. The accession number is GSE210386.
